# Evaluation of Android and Apple Store Depression Applications Based on Mobile Application Rating Scale

**DOI:** 10.3390/ijerph182312505

**Published:** 2021-11-27

**Authors:** Jaime Martín-Martín, Antonio Muro-Culebras, Cristina Roldán-Jiménez, Adrian Escriche-Escuder, Irene De-Torres, Manuel González-Sánchez, María Ruiz-Muñoz, Fermín Mayoral-Cleries, Attila Biró, Wen Tang, Borjanka Nikolova, Alfredo Salvatore, Antonio Cuesta-Vargas

**Affiliations:** 1 Department of Human Anatomy, Legal Medicine and History of Science, University of Málaga, 29071 Malaga, Spain; jaimemartinmartin@uma.es; 2 Instituto de Investigación Biomédica de Málaga (IBIMA), 29010 Malaga, Spain; amuro@uma.es (A.M.-C.); cristina.roldan@uma.es (C.R.-J.); adrianescriche@uma.es (A.E.-E.); mgsa23@uma.es (M.G.-S.); marumu@uma.es (M.R.-M.); fermin.mayoral.sspa@juntadeandalucia.es (F.M.-C.); 3 Department of Physiotherapy, University of Málaga, 29071 Malaga, Spain; 4 Physical Medicine and Rehabilitation Unit, Hospital Regional Universitary, 29010 Malaga, Spain; Irene.torres.sspa@juntadeandalucia.es; 5 Department of Nursing and Podiatry, University of Málaga, 29071 Malaga, Spain; 6 Mental Health Unit, Regional Universitary Hospital of Málaga, 29010 Malaga, Spain; 7 ITWare, 1117 Budapest, Hungary; attila.biro@itware.hu; 8 Faculty of Science and Technology, Bournemouth University, Bournemouth BH12 5BB, UK; wtang@bournemouth.ac.uk; 9 Arthaus, Production Trade and Service Company, 1000 Skopje, North Macedonia; borjanka@arthaus.mk; 10 Sensor ID Snc, 86021 Boiano, Italy; alfredo.salvatore@sensorid.it; 11 School of Clinical Sciences of the Faculty of Health, Queensland University of Technology, Brisbane 4000, Australia

**Keywords:** mobile application, depression, mobile health (mHealth), exercise, telemedicine, smartphone, mobile app rating scale (MARS)

## Abstract

There are a large number of mobile applications that allow the monitoring of health status. The quality of the applications is only evaluated by users and not by standard criteria. This study aimed to examine depression-related applications in major mobile application stores and to analyze them using the rating scale tool Mobile Application Rating Scale (MARS). A search of digital applications for the control of symptoms and behavioral changes in depression was carried out in the two reference mobile operating systems, Apple (App Store) and Android (Play Store), by means of two reviewers with a blind methodology between September and October 2019 in stores from Spain and the United Kingdom. Eighteen applications from the Android Play Store and twelve from the App Store were included in this study. The quality of the applications was evaluated using the MARS scale from 1 (inadequate) to 5 (excellent). The average score of the applications based on the MARS was 3.67 ± 0.53. The sections with the highest scores were “Functionality” (4.51) and “Esthetics” (3.98) and the lowest “Application Subjective quality” (2.86) and “Information” (3.08). Mobile Health applications for the treatment of depression have great potential to influence the health status of users; however, applications come to the digital market without health control.

## 1. Introduction

Depression is one of the great psychological diseases that occur throughout life. It has a prevalence close to 20%, being higher in women [1]. This produces a high number of hospital consultations and, therefore, a high expense in the public system, as well as being an important factor in people’s quality of life [2]. The sociodemographic and psychopathological factors and the course of the disease itself are fundamental in the treatment of this pathology, which allow one to determine whether it is a chronic or no chronic disorder [3].

The use of mobile phones as a tool for changing behavior and habits in patients is a reality. There are also a large number of mobile applications; in the Android Play store, there are 2,100,000 applications available, whereas there are 1,800,000 in the App Store [4]. The mobile applications used in health can be categorized into six main groups: lifestyle-oriented apps, patients-oriented apps, clinician-oriented apps, disease management systems, traditional telehealth, and mHealth systems [5]. However, not all applications, categorized as medical or health, have the same effect on patients [6].

Cognitive behavioral therapy (CBT) and behavioral activation are two of the most widely used evidence-based treatments for the treatment of depression, without considering pharmacological interventions [7]. However, the lack of adherence to these models and the lack of efficacy studies of them make the usefulness of mobile applications in this area questionable [8].

Users of different digital marketplaces can set scores from 1 to 5 for applications. This assessment is carried out completely subjective without establishing a prior criterion. This allows applications to place themselves in the highest positions in the search engine [9]. However, there are validated tools that allow the assessment of digital applications based on specific criteria [10], as well as assess the ability of the applications to produce behavioral changes [11]. However, users can access these applications freely without the guidance of a specialist. This fact does not ensure a reliable use of them, which can be harmful. In no case should these applications be a substitute for the intervention of a professional; they should be considered by users as a support tool [8].

The assessment of mobile applications can be carried out by means of the Mobile App Rating Scale (MARS) [10]. As far as we know, mobile applications for depression have not been assessed with this tool; it has been used for pain management [12], weight management [13], or asthma [14], among others. The MARS is a simple, objective, and reliable method to measure the quality of mobile applications, which has demonstrated good psychometric properties [10]. This instrument has been translated and adapted to Spanish while retaining its psychometric properties [15].

Therefore, it is of great relevance to carry out a systematic review of the mobile applications existing in the market focused on depression, assessing their user participation, and the functionality of the application, aesthetics, information provided, subjective quality, and impact of the application on the user based on the criteria established by the MARS. The results of this review will allow the specialist to recommend the best mobile applications for patients. Consequently, the aim of this study was to examine depression-related applications in major mobile application stores and analyze them using the rating scale tool MARS.

## 2. Materials and Methods

### 2.1. Information Sources and Search Strategy

This study included the mobile applications (apps) related to depression (free and paid) identified in the App Store (iOS) and Play Store (Android) in July 2019. Both systems are the most widespread among mobile phones; 99% of them use them. The search was conducted in the app stores of Spain and the UK.

Two searches were conducted, the first one with the term “depression” and the second one with the terms “depression” and “CBT” (cognitive behavioral therapy). The inclusion criteria for apps related to depression were: relation to include an evaluation system and to monitor the evaluations carried out. We also included those apps that implied behavior change techniques (BCTs) in the user or established objectives related to depression.

### 2.2. Eligibility Criteria

An initial evaluation was carried out that eliminated all “junk” apps that are not related to health problems, such as wallpaper apps or apps that only offer “text quotes.” Only one of the apps was analyzed in case these were repeated in different stores and only the language was changed (apps that were, for example, in the UK and Spanish Apple store). Apps related exclusively to depression assessment or general information, apps with a language other than English or Spanish, or apps that required an initial payment for its execution were also excluded.

### 2.3. Study Selection

**Figure 1:**
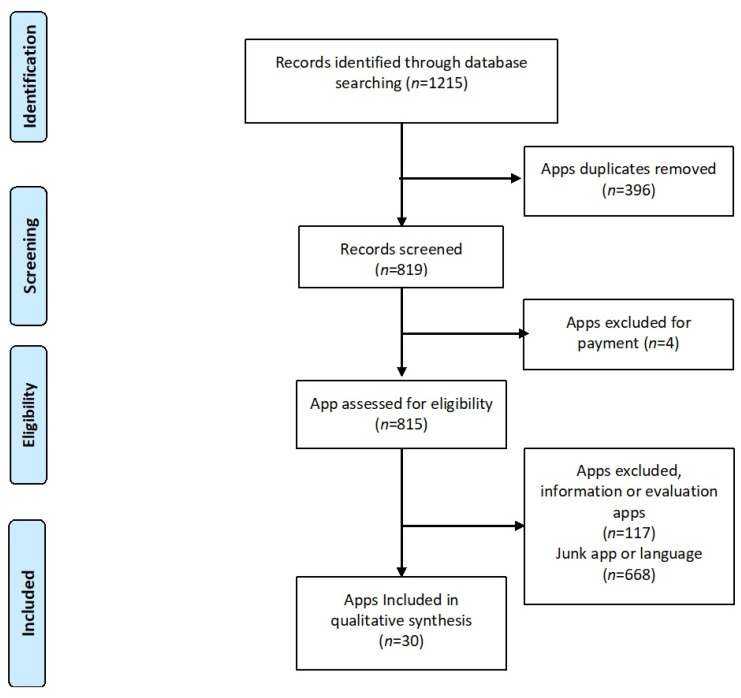
PRISMA Flow diagram for selected apps.

Two of the authors made the selection of apps based on the inclusion and exclusion criteria with a blind methodology. In case of conflict, a third author decided its inclusion or exclusion based on the established criteria. After performing the search, 30 apps were included (18 from the Android Play Store system and 12 from the Apple App Store operating system). A flow diagram based on PRISMA statement was included for the selected apps [16] (Figure 1). The included apps were independently assessed by two of the authors (J.M.-M. and A.M.-C) using the MARS Spanish assessment [15].

### 2.4. Data Extraction and Quality Assessment

The MARS has 23 structured questions in six sections: engagement, functionality, esthetics, information, app subjective quality, and app-specific. The questions are evaluated from 1 (inappropriate) to 5 (excellent); a final average score of the 4 initial sections is generated. The app subjective quality and app-specific sections are independently evaluated. MARS scores between the two reviewers were compared, and in case of discrepancies (two-point difference), they were compared. If there was still disagreement, a third reviewer participated to determine the score. The final score for each of the apps was obtained by the average of the scores of each reviewer.

### 2.5. Data Synthesis and Analysis

Statistical analysis was performed using SPSS statistical software, version 22.0 (SPSS Inc, Chicago, IL, USA). Average scores for each section of each app were retrieved. A descriptive analysis was performed for each of the MARS sections (mean and standard deviation). For inferential analysis, a bivariate correlation method was performed based on Pearson’s or Spearman’s coefficient according to the normality of the variables. The analyses carried out were: user ratings versus MARS ratings; app protection (binary variable) versus the number of downloads; app protection versus user scores; and app protection versus MARS.

## 3. Results

After an initial search for apps, a total of 1213 apps were found. A total of 30 apps (Figure 1) remained after the removal of paid apps, duplicated apps, and irrelevant apps. Eighteen applications from the Android Play Store and twelve from the App Store were included in this study. Likewise, 12 of the 30 applications (40%) included payments within the application to acquire full functionality or subscription. Only two of the included applications were not commercial affiliations (Table 1). 

All off the included apps (*n* = 30) were evaluated using the MARS tool for evaluating the quality of health apps (Table 2). No relationship was found between the number of downloads and the app’s MARS score r = 0.19 (*p* = 0.39). A moderate positive relationship was found between the users’ score and the MARS score as well, r = 0.48 (*p* = 0.04). No significant associations on the correlation analysis (*p* > 0.05) were shown between the app protection (binary variable) versus the number of downloads; app protection versus user scores; and app protection versus MARS. Based on the MARS results, the apps evaluated were divided into four quartiles (Table 2).

An overall score was obtained for each app covering the target fields of engagement, functionality, aesthetics, and information. The score of subjective subitems was also obtained. The best score based on the MARS was 4.58 for “Sanvello” and the lowest rating “PerSoNClinic (Depression, Chronic Pain, Cancer).“ Based on the engagement rating by the MARS, the best was “Savello.” For functionality, 10 apps obtained the best score; in esthetics, 3 applications rated the top score; in information, “MoodMission” was best; for app subjective quality, “WellTrack - Interactive Self-Help Therapy” and “Youper - Emotional Health“ were best; and for app-specific, 4 applications rated the best score (Table 3).

## 4. Discussion

In this paper, we aimed to use a tool commonly used for the analysis of mobile apps (MARS) to assess the quality of apps dedicated to depression. There are a multitude of these apps in the mobile device app stores, but in many cases, these apps are rated by the users who use them but lack an objective evaluation.

The MARS tool is a multidimensional tool that provides an overall score covering four objective quality indicators (engagement, functionality, aesthetics, and information quality) [10]. The MARS tool also has two other sections: one for subjective quality and a sixth section to assess the perceived impact of the app on the user. The score on the MARS scale ranges from 0 (worst) to 5 (best). Based on the results of our analysis, the “Sanvello” application obtained the highest results (4.58) and “Per-SoNClinic (Depression, Chronic Pain, Cancer)“ the lowest rating (2.38).

The initial search for apps was carried out in the official Google Play Store and Apple App Store in Spain and the UK. A total of 1213 apps were found in this initial search, of which a total of 30 apps were analyzed. It should be noted that for the apps to be included in this study, they had to be functional and related to the subject of depression. They also had to include a system for evaluating and monitoring the progress made.

It is worth noting that the market for mobile apps is constantly changing. Some apps are constantly updated or disappear, while new ones appear. In the course of this work, some apps that were found in the initial search had disappeared when we started to analyze them in detail.

In the case of the scores that the apps obtained from the users, these varied between 5 stars for the app with the highest score and 1.5 stars for the app with the lowest number of votes (average of 4.33). The apps obtained an average score on the MARS scale of 3.67, with scores ranging from 2.38 to 4.60. No correlation was observed between the users’ score and the score obtained on the MARS (*p* = 0.39). This type of result is not uncommon [10,17]. The apps that we can find in the app stores are usually commercial (in our sample, only one came from government and one from university). Users’ opinions are usually variable, unreliable, and subjective. This demonstrates the need to develop more science-based and less commercial apps in mHealth apps.

The tools most used for the management of depression in the apps analyzed were Assessment (18/30), CBT (17/30), and Feedback (15/30). CBT is a treatment approach for a range of mental and emotional health issues, and it is a technique widely used in mHealth apps [18].

A positive correlation was found between the number of downloads and the MARS score (*p* = 0.04). This could indicate that people do not just rely on user ratings, but also look for quality apps.

Nowadays, the issue of security is very important on the internet, even more so when dealing with personal health data [19,20], as is the case in the depression apps of this study. The MARS evaluates the safety of the app in two items: it asks if the app allows password protection and if it requires log-in. However, these items do not count in the overall MARS rating of the app. In the case of the apps seen in this study, only 50% met this minimum security (Table 1). Users do not seem to care much about privacy neither. No relationship was found between the security of the app and the number of downloads (Spearman’s rho = 0.27, *p* = 0.29), or the security of the app and user ratings (Spearman’s rho = 0.13, *p* = 0.56).

## 5. Conclusions

The use of smartphones is becoming more and more widespread. This leads to an increase in the use of apps among which mHealth apps stand out. mHealth apps (such as apps for the treatment of depression) have great potential to influence the health status of users who use them. Unfortunately, most of the apps we found in app stores have a commercial purpose with a lack of scientific rigor. These apps come to the market from mobile app stores without any kind of health control. The only control they have is the publication policy of the app store itself. In many cases, we have found apps that only showed some information about depression (without any information about where it was obtained), forums for people with depression (without any control over the forum), or that gave access to an online psychologist. Not only could some of these apps not be beneficial for depression, but in some cases could be harmful to the individual. This shows the great importance of developing apps with scientific rigor that can bring benefits to the individual who uses them.

## Figures and Tables

**Table 1:** Description of the apps included in the study.

App Name	Platform	Password Protected	Version	Paid Content	Downloads	Affiliations
BoosterBuddy	iOS	NO	1.7	Free	N/A	Government
Cath It—Make sense of moods	iOS	YES		Free	N/A	University
Flow Depression	iOS	YES	1.1.5	Free	N/A	Commercial
MindShift CBT Anxiety Canada	iOS	NO	2.0.4	Free	N/A	Commercial
Overcoming -Depression	iOS	NO	1.1	Free	N/A	Commercial
What’s Up? A mental Health App	iOS	NO	2.3.1	Free	N/A	Commercial
DBT Coach	iOS	NO		Free	N/A	Commercial
Happier You	iOS	NO	2.0.8	Free	N/A	Commercial
MoodMission	iOS	YES	1.1	Free	N/A	Commercial
Simple DBT Skills Diary Card	iOS	NO	1.4.1	Free	N/A	Commercial
Happy Being	iOS	YES	4.1	Free	N/A	Commercial
Super Better	iOS	YES	1.7	Free	N/A	Commercial
InnerHour—Self Help for Anxiety and Depression	Android	YES	3.16	Free	+100,000	Commercial
Moodpath—Depression and Anxiety Test	Android	NO	2.1.2	In-app purchases	+500,000	Commercial
MoodTools—Depression Aid	Android	NO	2.4.4	In-app purchases	+100,000	Commercial
Sanvello	Android	YES	8.1.0	In-app purchases	+1,000,000	Commercial
UP!—El Diario Anímico para Personas Bipolares	Android	YES	1.5.11	Free	+50,000	Commercial
Woebot: Your Self-Care Expert	Android	YES	3.6.1	In-app purchases	+100,000	Commercial
Wysa: tu entrenador de felicidad	Android	YES	1.0.2	In-app purchases	+1,000,000	Commercial
Diario de animo y preguntas (anti depresión)	Android	NO	1.2.12	In-app purchases	+100,000	Commercial
PerSoNClinic (Depression, Chronic Pain, Cancer)	Android	NO	2.1	Free	+1000	Commercial
UpLift for Depression	Android	YES	1.1.3	In-app purchases	+1000	Commercial
GG Depression: Quick daily exercise	Android	NO	2.01.69	In-app purchases	+1000	Commercial
Lift – Depression and Anxiety	Android	NO	1.6.39	In-app purchases	+100	Commercial
Moodfit—Stress and Anxiety	Android	YES	2.9.4	Free	+5000	Commercial
WellTrack—Interactive Self-Help Therapy	Android	YES	2.9.5	In-app purchases	+10,000	Commercial
Youper—Emotional Health	Android	YES	6.11.001	In-app purchases	+500,000	Commercial
What’s Up?—Mental Health App	Android	NO	2.3.3	In-app purchases	+100,000	Commercial
Appyness Healthwatch	Android	YES	1.1	Free	+100	Commercial
WellMind	Android	NO	1.0.2	Free	+10,000	Commercial

N/A: Not available.

**Table 2:** Theoretical background/strategies and focus of the app (according to the MARS scales).

App Name	Focus	Theoretical Background/Strategies
BoosterBuddy	Depression, Anxiety/Stress	Advice/Tips/Strategies/Skills training
Cath It—Make sense of moods	Depression, Anxiety/Stress, Behaviour change	CBT—Behavioural (positive events)
Flow Depression	Depression, Anxiety/Stress, Behaviour change	Monitoring/Tracking, Advice/Tips/Strategies/Skills training, CBT—Cognitive (thought challenging), Mindfulness/Meditation
MindShift CBT Anxiety Canada	Anxiety/Stress, Behaviour change	CBT—Cognitive (thought challenging)
Overcoming -Depression	Behaviour change	CBT—Cognitive (thought challenging)
What’s Up? A mental Health App	Depression, Anxiety/Stress	CBT—Cognitive (thought challenging)
DBT Coach	Reduce negative emotions, Depression, Anxiety/Stress	CBT—Cognitive (thought challenging), CBT—Behavioural (positive events)
Happier You	Reduce negative emotions, Depression, Anxiety/Stress	CBT—Cognitive (thought challenging)
MoodMission	Mindfulness/Meditation/Relaxation, Reduce negative emotions, Depression, Anxiety/Stress	CBT—Cognitive (thought challenging), CBT—Behavioural (positive events)
Simple DBT Skills Diary Card	Reduce negative emotions, Depression, Anxiety/Stress	Feedback
Happy Being	Reduce negative emotions, Depression, Anxiety/Stress	CBT—Behavioural (positive events), CBT—Cognitive (thought challenging)
Super Better	Reduce negative emotions, Depression, Anxiety/Stress	CBT—Cognitive (thought challenging)
InnerHour—Self Help for Anxiety and Depression	Increase happiness/Well-being, Depression, Anxiety/Stress, Anger, Behaviour change	Assessment, Feedback, CBT—Behavioural (positive events)
Moodpath—Depression and Anxiety Test	Increase happiness/Well-being, Reduce negative emotions, Depression, Anxiety/Stress.	Assessment, Feedback, CBT—Behavioural (positive events)
MoodTools—Depression Aid	Reduce negative emotions, Depression, Anxiety/Stress.	Assessment, Feedback
Sanvello	Increase happiness/Well-being, Depression, Anxiety/Stress.	Assessment, Feedback, Information/Education, CBT—Behavioural (positive events)
UP!—El Diario Anímico para Personas Bipolares	Reduce negative emotions, Depression, Anxiety/Stress.	Assessment, Feedback
Woebot: Your Self-Care Expert	Increase happiness/Well-being, Reduce negative emotions, Behaviour change	Assessment, Feedback, CBT—Behavioural (positive events)
Wysa: tu entrenador de felicidad	Increase happiness/Well-being, Mindfulness/Meditation/Relaxation, Reduce negative emotions, Depression, Behaviour change	Assessment, Feedback, Information /Education, CBT—Behavioural (positive events), Advice /Tips /Strategies /Skills training
Diario de animo y preguntas (anti depresión)	Reduce negative emotions	Assessment, Feedback
PerSoNClinic (Depression, Chronic Pain, Cancer)	Depression, Alcohol /Substance use	Assessment, Feedback, Information /Education
UpLift for Depression	Increase happiness/Well-being, Reduce negative emotions, Depression, Anxiety/Stress	Assessment, Feedback, Information /Education, CBT—Behavioural (positive events)
GG Depression: Quick daily exercise	Increase happiness/Well-being, Reduce negative emotions, Depression	Assessment, Feedback
Lift—Depression and Anxiety	Increase happiness/Well-being, Reduce negative emotions, Depression	Assessment, Feedback, Monitoring/Tracking
Moodfit—Stress and Anxiety	Increase happiness/Well-being, Reduce negative emotions, Depression, Behaviour change, Goal setting	Assessment, Feedback, Monitoring/Tracking, Goal setting, CBT—Behavioural (positive events)
WellTrack—Interactive Self-Help Therapy	Increase happiness/Well-being, Mindfulness/Meditation/Relaxation, Reduce negative emotions, Depression, Anxiety/Stress, Behaviour change	Assessment, Information/Education, Feedback, Monitoring/Tracking, Advice /Tips /Strategies /Skills training
Youper—Emotional Health	Increase happiness/Well-being, Reduce negative emotions, Depression, Anxiety/Stress, Goal setting	Assessment, Feedback, Information/Education, Monitoring/Tracking, Goal setting
What’s Up?—Mental Health App	Increase happiness/Well-being, Reduce negative emotions, Depression, Anxiety/Stress, Behaviour change	Assessment, Information/Education, Monitoring/Tracking
Appyness Healthwatch	Increase happiness/Well-being, Reduce negative emotions, Depression, Anxiety/Stress, Behaviour change	Assessment, Information/Education, Monitoring/Tracking, Advice /Tips /Strategies /Skills training
WellMind	Increase happiness/Well-being, Reduce negative emotions, Depression, Anxiety/Stress	Assessment, Information/Education, Monitoring/Tracking, Advice /Tips /Strategies /Skills training

CBT: Cognitive Behavioral Therapy.

**Table 3:** Mobile Application Rating Scale of applications.

App Name	User Ratings in the Store (Votes)	App Quality Mean Score for MARS Sections							App Quality Mean Score
		Q ª	Engagement	Functionality	Esthetics	Information	App Subjective Quality	App Specific	
BoosterBuddy	5.0 (1)	Q4	2.60	4.75	3.67	2.57	2.25	1.83	3.40
Cath It—Make sense of moods	N/A	Q4	2.40	5.00	3.67	2.29	2.00	2.33	3.34
Flow Depression	N/A	Q1	3.80	5.00	4.00	3.86	3.25	4.17	4.16
MindShift CBT Anxiety Canada	4.8 (4)	Q2	3.60	5.00	4.33	2.29	2.50	3.17	3.80
Overcoming -Depression	N/A	Q4	1.20	4.75	3.33	1.86	1.75	2.50	2.79
What’s Up? A mental Health App	5.0 (2)	Q3	2.80	4.75	5.00	2.14	3.00	3.33	3.67
DBT Coach	N/A	Q1	4.00	5.00	4.33	3.57	3.50	4.50	4.23
Happier You	N/A	Q2	3.00	5.00	4.33	3.14	3.00	3.83	3.87
MoodMission	N/A	Q2	3.00	4.50	4.00	4.43	4.25	5.00	3.98
Simple DBT Skills Diary Card	N/A	Q3	2.00	4.50	4.67	3.14	2.00	2.33	3.58
Happy Being	1.5 (2)	Q4	2.40	3.00	2.67	2.43	1.25	1.83	2.62
Super Better	4.5 (27)	Q2	3.40	5.00	3.33	3.71	3.75	3.83	3.86
InnerHour—Self Help for Anxiety and Depression	4.5 (3000)	Q1	4.00	4.75	4.00	3.29	3.25	3.33	4.01
Moodpath—Depression and Anxiety Test	4.6 (15,000)	Q3	3.20	4.50	3.67	3.43	3.25	3.17	3.70
MoodTools—Depression Aid	4.4 (2962)	Q4	2.80	4.00	2.67	2.43	1.75	2.50	2.97
Sanvello	4.4 (12,486)	Q1	4.60	4.75	4.67	4.29	4.25	4.33	4.58
UP!—El Diario Anímico para Personas Bipolares	4.5 (1000)	Q4	2.80	3.75	3.67	3.00	2.00	2.00	3.30
Woebot: Your Self-Care Expert	4.6 (4343)	Q2	3.40	4.50	3.33	3.71	3.75	3.67	3.74
Wysa: tu entrenador de felicidad	4.5 (20,074)	Q2	3.60	4.75	4.33	3.29	3.75	3.67	3.99
Diario de animo y preguntas (anti depresión)	4.7 (1288)	Q3	3.20	4.50	4.00	2.86	2.25	2.00	3.64
PerSoNClinic (Depression, Chronic Pain, Cancer)	4.6 (10)	Q4	3.00	1.75	2.33	2.43	1.00	2.00	2.38
UpLift for Depression	N/A	Q3	3.20	4.25	3.67	3.00	1.75	2.33	3.53
GG Depression: Quick daily exercise	4.3 (22)	Q2	2.60	5.00	4.67	3.00	3.25	4.50	3.82
Lift – Depression and Anxiety	4.2 (8)	Q3	3.20	3.50	4.00	3.00	3.50	5.00	3.43
Moodfit—Stress and Anxiety	4.3 (118)	Q1	3.80	4.50	5.00	3.71	4.25	4.50	4.25
WellTrack—Interactive Self-Help Therapy	3.1 (45)	Q1	3.80	5.00	4.67	3.57	4.50	5.00	4.26
Youper—Emotional Health	4.9 (37,965)	Q1	4.40	5.00	5.00	4.00	4.50	5.00	4.60
What’s Up?—Mental Health App	4.4 (3028)	Q3	3.60	4.75	4.00	2.43	2.25	2.50	3.69
Appyness Healthwatch	5.0 (2)	Q4	1.80	4.75	3.67	1.71	1.50	1.83	2.98
WellMind	3.4 (91)	Q2	2.00	5.00	4.67	3.71	2.50	3.17	3.85
Total Mean (SD)	4.33 (0.78)		3.11 (0.78)	4.51 (0.71)	3.98 (0.69)	3.08 (0.71)	2.86 (1.02)	3.31 (1.09)	3.67 (0.53)

N/A: Not Available; Q ª: Quartile; SD: Standard Deviation.
